# A Sensor for Multi-Point Temperature Monitoring in Underground Power Cables

**DOI:** 10.3390/s25175490

**Published:** 2025-09-03

**Authors:** Pedro Navarrete-Rajadel, Pedro Llovera-Segovia, Vicente Fuster-Roig, Alfredo Quijano-López

**Affiliations:** 1 Navarrete Ingenieros, 46017 Valencia, Spain; pedro@navarreteingenieros.com; 2 Instituto de Tecnología Eléctrica, Universitat Politècnica de València, 46022 Valencia, Spain; vfuster@ite.upv.es (V.F.-R.); alfredo.quijano@ite.es (A.Q.-L.); 3 Instituto Tecnológico de Energía, 46980 Paterna, Spain

**Keywords:** temperature measurement, underground cables, NTC thermistors, distributed sensor, electrical distribution networks

## Abstract

Underground electrical conductors, both medium-and high-voltage, play a crucial role in energy infrastructure. However, they present a maintenance challenge due to their difficult access. Unlike overhead installations, these cables remain hidden, making it harder to obtain key parameters, such as their temperature or structural condition, in a simple manner. Current temperature measurement methods, including fiber-optic-based systems (DTS and LTS), involve high costs that limit their feasibility in medium-voltage networks, where more economically accessible alternatives are required. This study introduces an alternative system for monitoring the temperature of underground cables using NTC thermistors. Its design allows for reducing the number of connection conductors for sensors to just four regardless of the number of measurement points. The implemented measurement technique is based on the sequential activation of sensors and the integration of the recorded current to achieve an accurate thermal assessment. The tests conducted validate that this proposal represents an efficient, cost-effective, and highly scalable solution for implementation in electrical distribution networks.

## 1. Introduction

Underground medium- and high-voltage cables are critical infrastructure for the electric power network. Effective management of these assets requires in-depth knowledge of several key operational parameters, most notably their real-time current-carrying capacity (ampacity) and their long-term ageing condition [1]. On the one hand, accurate ampacity estimation is crucial for optimizing load distribution, maximizing the dynamic ampacity ratings, allowing operators to optimize the energy transport capacity of the cable without the risk of exceeding its thermal operating limits [2,3,4]. On the other hand, assessing the ageing state of these cables is fundamental for asset management, enabling utilities to plan maintenance and replacement activities with foresight and cost efficiency. Thus, two interrelated but temporally distinct questions must be addressed: (1) what is the cable’s current ampacity in real time [5], and (2) what is its ageing condition to support medium- and long-term infrastructure planning?

Cable temperature monitoring offers a pathway to answer both questions, as cable temperature is directly related to both electrical loading and thermal ageing [6]. However, in underground medium- and high-voltage lines, temperature measurement is a difficult task because cables are, in general, not accessible. And it is especially difficult in medium-voltage lines due to the complexity of the network and its large dimensions. Consequently, novel and practical solutions are required to overcome these limitations and enable reliable temperature-based diagnostics.

**Figure 1:**
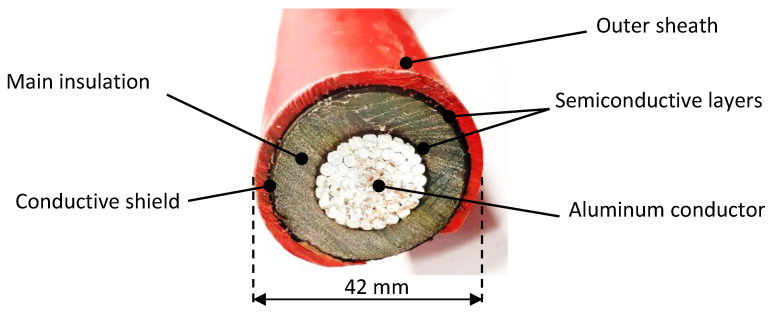
Cross-sectional image of a 20 kV underground cable.

Underground cables (see Figure 1) are characterized by having a multilayer structure. The high-voltage potential is on the inner conductor, which is isolated by the main insulation layer. The electric field is confined between the inner conductor and the conductive shield, which is grounded. To ensure a uniform electric field distribution and to avoid stress concentrations at material interfaces, semiconductive layers are applied both over the conductor and beneath the shield. An outer sheath protects them from environmental conditions.

Heat generation in cables arises from electrical conduction losses. Even though the conductors are made of low-resistivity materials such as copper or aluminum, the flow of current induces heating due to the Joule effect. The power dissipated as heat, $P_{Heat}$, is proportional to the electrical resistance, $R_{cable}$, and the square of the current amplitude, i, according to the following well-known relation:

(1) $$P_{Heat}=R_{cable}i^{2}$$

In alternating current (HVAC) cables, additional heat is generated by capacitive currents and by induced currents in the conductive shield, and to a lesser extent by dielectric losses in the main insulation layer. In contrast, direct current (HVDC) cables do not experience induced currents in the shield, and dielectric losses are significantly lower.

The heat generated in the conductors must be dissipated through the cable structure and into the surrounding environment, but its high thermal resistance causes the temperature to rise more than, for example, in bare overhead conductors directly exposed to air. Although high thermal resistivity of electrical insulating materials is not a characteristic specifically targeted during the design process, it is a natural consequence of using electrical insulating materials, which inherently have low thermal conductivity.

An excessive conductor temperature can degrade the insulation and reduce the cable’s lifespan, making the thermal limit of the cable directly dependent on the properties of the insulating material. Insulating materials in underground cables are polymers that exhibit low maximum operating temperatures compared to metals. Typical temperature limits for polymer-insulated cables range around 70 °C, 90 °C, or even 110 °C, depending on the material. Beyond these temperatures, the cable insulation degrades more rapidly. Therefore, for the ampacity determination of a cable, the maximum current-carrying capacity is limited by the temperature limit of the insulation.

The maximum temperature in a power cable is reached at the inner conductor. However, direct measurement of the conductor temperature is not feasible in practice, as it would require contact with high-voltage components. As a result, temperature sensors are typically placed on the outer sheath, and the conductor temperature must be estimated indirectly using a thermal model of the cable [7,8]. This thermal model must account for all the layers of the cable structure and must also incorporate real-time current measurements [9,10,11,12]. When the temperature is measured at the surface of the sheath, the complexity of the model is significantly reduced, as it becomes possible to relate the surface temperature to the conductor temperature through a known thermal gradient provided the cable geometry and material properties are well characterized.

In addition to its role in determining ampacity, temperature is also a critical variable in lifetime prediction models for underground cables. Since the pioneering work of Dakin [13], cable ageing has been generally treated as a degradation process that is thermally triggered. Several models have been developed based on the temperature that cables have experienced during their operation in combination with the electric field [6,13,14,15]. These models rely on knowing the cable temperature to estimate the degree of degradation and determine the theoretical remaining life. A cable that has spent most of its life at temperatures below 60 °C will have aged less than one that has regularly operated above 70 °C, even if it has never exceeded its critical temperature. Thermal ageing is a cumulative process that can be estimated through temperature monitoring.

From both perspectives of real-time ampacity and cable life models for asset management, temperature is a key parameter. In high-voltage cables (typically above 110 kV), temperature monitoring systems are frequently employed because of the criticality of some lines. Measurement is performed by means of distributed temperature sensing (DTS) systems, which are based on optical fiber technology. DTS systems allow for continuous and spatially resolved temperature monitoring along the entire length of the cable. They can provide accurate temperature profiles of the cable sheath and they can also detect hotspots [5]. The optic fiber can be attached externally to the cable or embedded within the sheath during the manufacturing process.

While DTS systems have proven highly effective in high-voltage applications, they are generally not economically viable for medium-voltage (MV) networks, which are significantly more extensive, less accessible, and lower in both cost and criticality. Recently, a proposal was made to use DTS on MV networks that demonstrated that it is technically feasible, although cost estimations were not provided [16]. A new approach based on the Frequency Response Analysis (FRA) combined with Machine Learning techniques allows the detection of overheated cable sections through impedance variations caused by temperature changes [17]. Although this method does not provide precise temperature measurements, it can identify hotspots with a certain degree of spatial resolution. In [18], the average temperature of a monitored cable equipped with an on-line partial discharge measurement system was indirectly inferred by analyzing the propagation speed of an injected pulse used for system self-calibration and synchronization. This method allows for average temperature monitoring but cannot detect localized hotspots. A similar strategy is presented in [19], where the electromagnetic time reversal technique was applied to determine the average propagation speed in a cable, thereby estimating its average temperature.

In fact, within the 20 kV distribution networks commonly found across Europe, the actual temperature profile of underground cables, or even their average temperature, remains largely unknown due to the lack of continuous monitoring systems.

Proposals have been made for measurement systems at a finite number of points, also known as localized temperature sensing (LTS) [20], which is also based on fiber optics. In this case, its economic feasibility for medium-voltage distribution networks remains uncertain. Although the exact cost is not well established, it is expected to be relatively high due to the nature of the underlying technology. As an alternative, ref. [21] describes a more affordable fiber-based system designed to measure the temperature of joints in underground cables, trading off accuracy in favor of significantly reduced system costs.

A potentially economical solution for an LTS system is the well-known DS18B20 technology (Analog devices, Wilmington, MA, USA), which provides the so-called one-wire temperature measurements [22] using a thermistor as the sensing element. This is a popular solution compatible with commercial microprocessors such as the Arduino. The system employs a three-wire configuration: two wires for the sensor power supply and a third for communication, shared with the ground wire. Due to the characteristics of the communication signal, the maximum recommended cable length for a DS18B20 system using a single microcontroller is approximately 100 m, based on user experience. Additionally, the number of sensors is constrained by the overall cable length. It is generally recommended not to exceed 25 sensors for short cables and to use 10 or fewer for cables longer than 30 m. While this system may be suitable for monitoring short sections of underground cable, it is not applicable to typical underground cable installations, which often exceed 100 m and may span several kilometers.

Therefore, a solution is needed that allows for temperature monitoring in MV underground cables while being economically viable for installation across thousands of cables in distribution networks. If a measurement of the temperature distribution in a cable with high spatial resolution is not feasible, then at least a representative amount of measurement points should be obtained. An LTS system with a high number of measurement points and that is feasible for underground medium-voltage networks could be a trade-off solution. In this paper, we propose an LTS system based on thermistors as the sensing element. The system requires four wires and supports a high number of measurement points (up to 50 or more) without being limited by cable length, as temperature measurements are obtained via a current loop. The system relies on low-cost electronics and provides sufficient accuracy for temperature monitoring in MV underground cables.

## 2. Materials and Methods

### 2.1. General Structure of the Measuring System

The proposed solution allows for temperature measurement on the cable sheath at a discrete number of locations spaced either at regular or arbitrary intervals. Unlike distributed temperature sensing (DTS), this approach does not provide a continuous temperature profile, but it offers a practical compromise between cost, complexity, and data relevance. The system is not technically limited by the cable length but rather by a maximum number of measurement points. The architecture is modular and scalable; the number of sensing points could be extended to 100 points, or even 200 or more if two measurement systems are installed from each end of an underground cable. Each system would monitor approximately half of the cable length.

The solution is based on the use of thermistors as the sensing element. Thermistors are resistive components whose electrical resistance varies with temperature. In this system, negative temperature coefficient (NTC) thermistors are used. To determine the temperature at each measurement point, a constant voltage, V, is applied across the thermistor, and the resulting current, i, is measured. Using Ohm’s Law, the resistance of the thermistor can be calculated as

**Figure 2:**
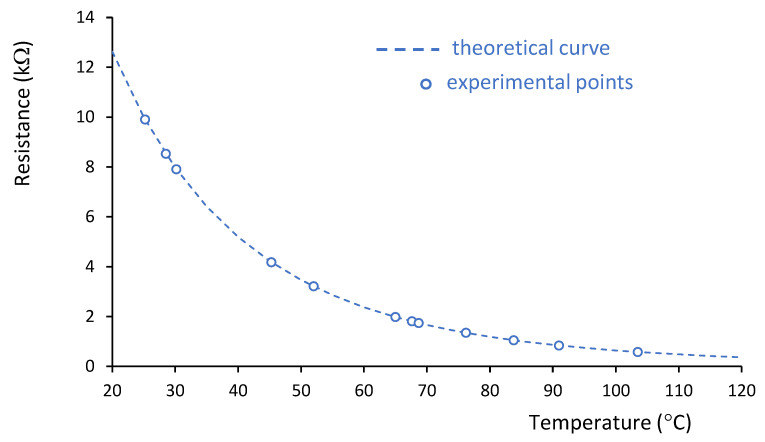
Example of the resistance variation curve of a thermistor as a function of temperature.

(2) V=R(T)×i

where R(T) is the resistance, which depends on the component’s temperature. This is a well-known method for temperature measurement. The variation of R(T) with temperature follows a logarithmic pattern. In our system, 10 kΩ NTC thermistors with a time constant of 10 s have been chosen. Their characteristic curve resistance vs temperature has been experimentally verified, as shown in Figure 2.

**Figure 3:**
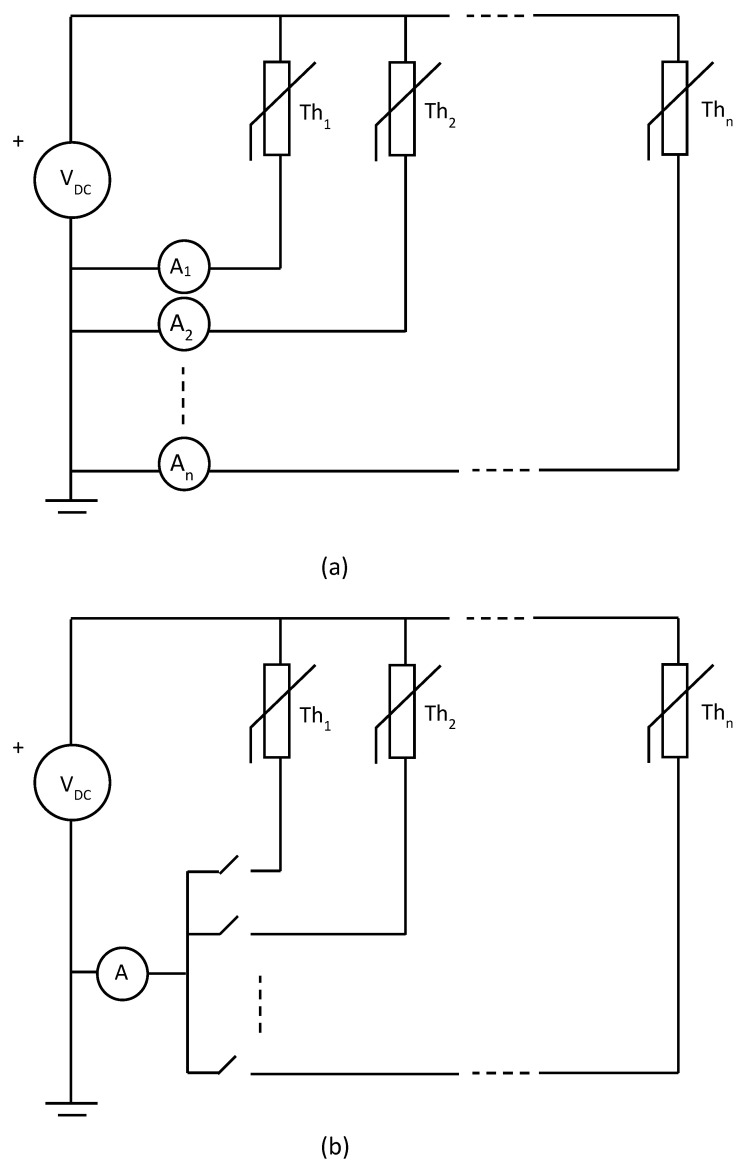
Structure of a measuring system using n thermistors and n + 1 wires: (**a**) with n independent current measurement circuits; (**b**) with a multiplexed current measurement circuit.

The proposed cable temperature measurement system is based on placing NTC thermistors along the cable, with flexible spacing that can be adapted to the specific monitoring requirements of each installation. In a direct measurement scheme, each thermistor would require its own current-sensing circuit, leading to a system that needs n + 1 wires (for n sensors) and n current measurement channels. This approach quickly becomes impractical as the number of measurement wires and sensors increases, as shown in Figure 3a. To address this limitation, the system can adopt a multiplexed current measurement architecture, where a single current-sensing circuit is shared among multiple thermistors through an electronic switching mechanism. This approach reduces the amount of measurement equipment required while maintaining measurement functionality. However, it still preserves the n + 1 wire structure, as shown schematically in Figure 3b. A short delay between readings is introduced due to the sequential acquisition, although this is not relevant for slow-varying thermal measurements, such as those typically encountered in underground cable monitoring.

**Figure 4:**
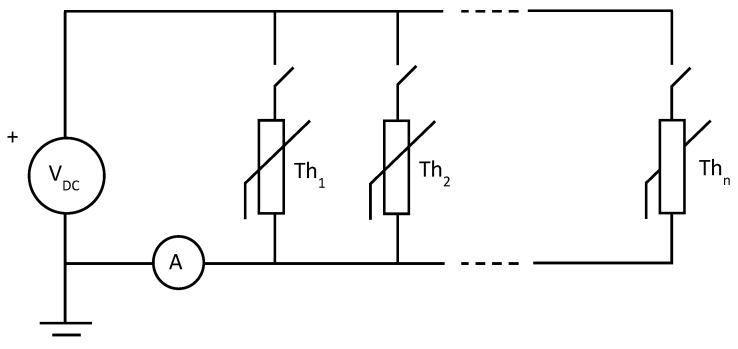
Structure of a measuring system of n thermistors sequentially activated.

The proposed solution is based on sequentially activating each thermistor and measuring the total current. This reduces the total number of wires. Only one current measurement system is required (Figure 4).

**Figure 5:**
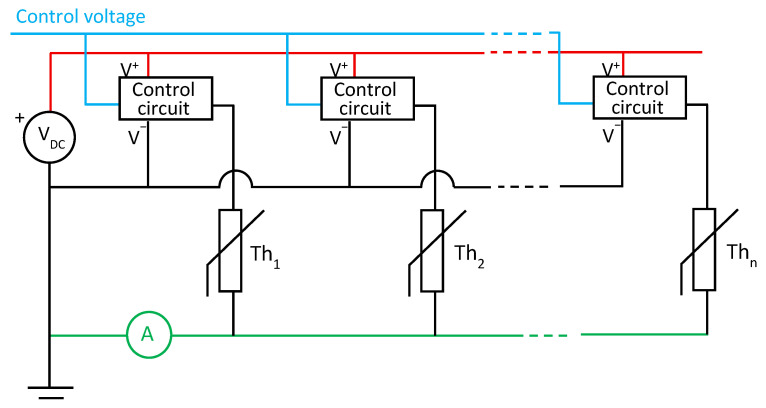
Structure of a measuring system of n thermistors with a control unit and a 4-wire system.

To achieve the sequential activation of thermistors, each thermistor is equipped with a small control circuit activated remotely by a control voltage. Thus, only four wires are required (Figure 5):

- Two wires for powering control circuit.
- One wire for a control signal.
- One wire for current measurement.

At the beginning of each measurement cycle, no voltage is applied to the thermistors. The thermistor is in thermal equilibrium with the cable sheath. When a temperature measurement is needed, a voltage is applied to the thermistor and the resulting current is measured. This measurement procedure must be fast enough to avoid thermistor self-heating.

Two activation strategies are possible:

- In the first, only one thermistor is activated at a time, and the measured current corresponds solely to that sensor. This approach simplifies the interpretation of the measurement, but it requires a more complex control circuit to activate individual thermistors.
- In the second, thermistors are activated sequentially without deactivating the previous ones so that the measured total current increases in steps. Each step corresponds to the current contribution of the most recently activated thermistor. This method simplifies the hardware, but it requires postprocessing of the current signal to extract the individual readings.

In our implementation, we adopted the second strategy, which results in reduced hardware complexity and smaller system size while still enabling accurate temperature measurement. However, both methods are viable.

To reduce power consumption, all the control circuits are powered down until a measurement is required. When the measurement starts, the lines V^+^ and V^−^ in Figure 5 are energized first (thermistors are still not powered). Then, the control voltage is gradually increased, and each control circuit activates its thermistor when a voltage threshold is reached.

Although this system uses 4 wires instead of the 3 wires used by the DS18B20 (Analog devices, Wilmington, MA, USA) the measurement signal is based on a DC current loop rather than a digital communication interface. This approach enables long-distance measurements with virtually no signal attenuation. Additionally, the number of sensors is not constrained by communication issues or cable length. As will be discussed below, the system’s limitations are instead determined by the control voltage discrimination required for the individual activation of thermistors and by the characteristics of the current measurement device.

### 2.2. Single Measurement Cell Description

**Figure 6:**
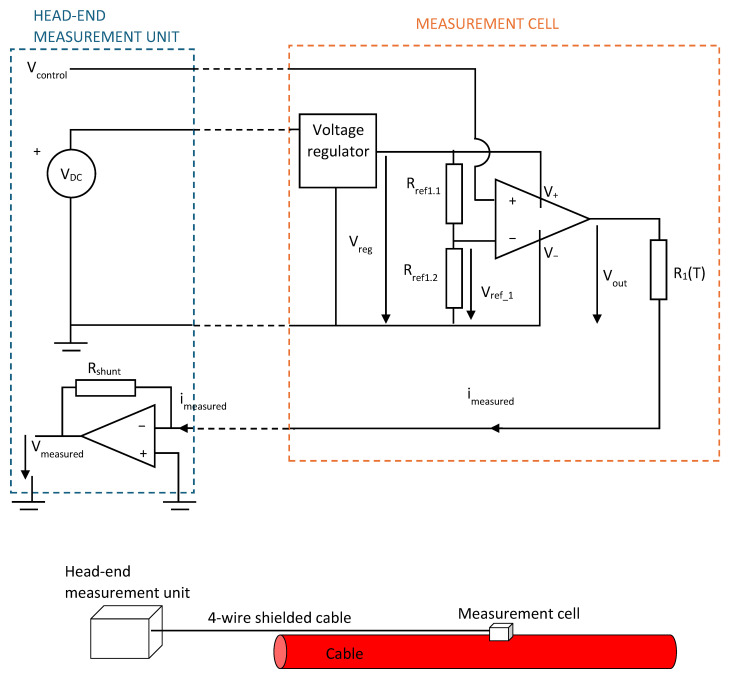
Diagram of the basic measurement unit of the system.

To illustrate the proposed system in detail, a single measurement cell will be described first. The electronic schematic and the spatial arrangement of the elements along the cable are shown in Figure 6. The system is composed of two main components: (1) the measurement cell, which is located at a specific point along the cable, and (2) the head-end measurement system, which provides power and the control signal and which performs the acquisition and processing of the current signals.

Each measurement cell includes the sensing element (NTC thermistor), switching electronics for activation, and minimal circuitry to interface with the shared supply and control lines.

To ensure the voltage applied to the thermistor, a voltage regulator circuit is used next to it, so that the following condition is always met:

(3) $$V_{DC}>V_{reg}$$

Typical values can be V_DC_ = 20 V and V_reg_ = 15 V.

The control voltage, V_control_, is received from the head-end measurement unit, which is typically installed in the secondary substation. The functions of the head-end measurement system can be performed using standard components, such as microcontrollers, computers, and PLCs, or specifically designed circuits. In our implementation, we used a computer-based setup composed of a National Instruments (Austin, TX, USA) USB data acquisition card and a LabVIEW^TM^ 2014 (National Instruments) application to generate the control loop and the control voltage and to acquire and analyze the measured current.

In the measurement cell, the control voltage is compared to a voltage reference, V_ref_1_. This reference voltage is generated locally using a resistive voltage divider composed of resistors R_ref1.1_ and R_ref1.2_.

When V_control_ exceeds V_ref_1_, the comparator switches to a saturated state, and the output voltage V_out_ changes from 0 V to the saturation voltage value V_sat_ (slightly lower than V_reg_ but of known magnitude).

(4) $$V_{control}>V_{ref\_1}$$

At that moment, a current i_measured_ is established in the circuit with the following value:

(5) $$i_{measured}=\frac{V_{sat}}{R(T)}$$

In this equation, the wire resistance is neglected since the resistance of the thermistor, R(T), is in the range of several kΩ. The current is independent of the shunt resistance due to the virtual ground at the inverting input of the operational amplifier. The output voltage V_measured_ is given by

(6) $$V_{measured}={-R}_{shunt}i_{measured}$$

By combining (4) and (5), the value of R(T) can be calculated:

(7) $$R\left(T\right)=\frac{V_{sat}}{i_{measured}}=-\frac{V_{sat}}{V_{measured}}R_{shunt}$$

Finally, using the equation of the thermistor (Figure 2), the temperature value is obtained. As shown in the system layout, the connection cable between the head-end measurement system and the measurement cell consists of four wires: two for power supply (V^+^ and V^−^), one for the control voltage (V_control_), and one for current measurement. For reliable operation, especially in environments with electrical noise or electromagnetic interference, it is recommended that the cable includes shielding to protect the integrity of the measurement signals.

If additional measurement cells are to be added along the cable, the same 4-wire connection can simply be extended to include the new units, as will be described in the following section. This modularity allows for straightforward scaling of the monitoring system.

### 2.3. Multiple Cell Measurement System

The structure described for a single measurement cell can be extended to an array of n measurement cells distributed along the cable. In the implemented solution, the control system activates the measurement cells sequentially, causing their contributions to the total measured current to accumulate progressively. That is, initially only cell 1 conducts, then cells 1 and 2 conduct simultaneously, then cells 1, 2, and 3, and so on until N measurement points are conducting. This behavior is achieved by defining the reference voltages of each cell, V_ref_i_, which are unique for each cell, in ascending order. By progressively increasing the control voltage, V_control_, from the head-end system, each cell begins conducting sequentially.

The limit of measurement points is fundamentally constrained by the ability to individually activate each cell. This depends on the number of distinct control levels that can be unambiguously defined and on the reliability detected by the threshold circuits to activate each cell individually. In our system, a 200 mV margin was maintained between reference voltages. With a 10 V control signal, up to 50 measurement cells can be controlled. Using control signals with higher amplitude (for example, 30 V) and improving the precision of voltage threshold detection, it is possible to increase the number of measurement points.

Alternatively, the system could be configured so that each measurement cell is turned on and off individually using a control window defined by two voltage thresholds: one to activate and another to deactivate the cell. This strategy would ensure that only one thermistor is active at any given time, simplifying current interpretation. However, this approach requires two thresholds per thermistor, effectively halving the number of usable control levels and reducing the total number of measurement cells that can be supported within a given voltage range. Additionally, it introduces slightly increased complexity in the control circuitry.

**Figure 7:**
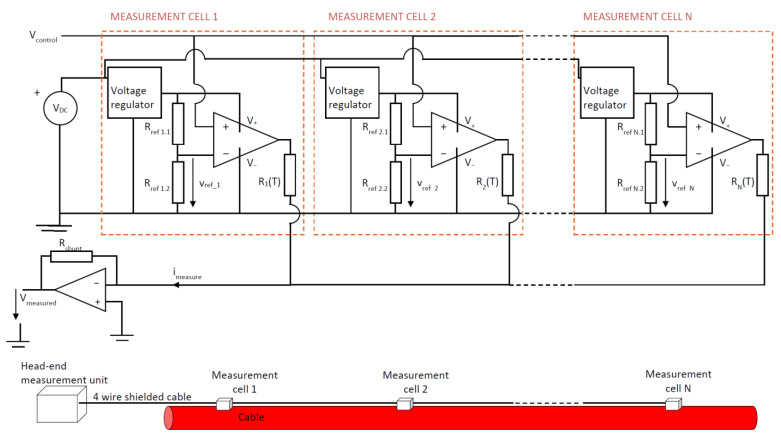
Stepped voltage control for n-cells system.

The basic connection scheme is shown in Figure 7. The supply voltage, V_DC_, and the control voltage, V_control_, are distributed to all the measurement cells. The current flowing through the activated thermistors is collected in a common return wire, which carries the current toward a measuring circuit located in the head-end unit. This configuration allows the entire measurement system to operate using only four wires.

**Figure 8:**
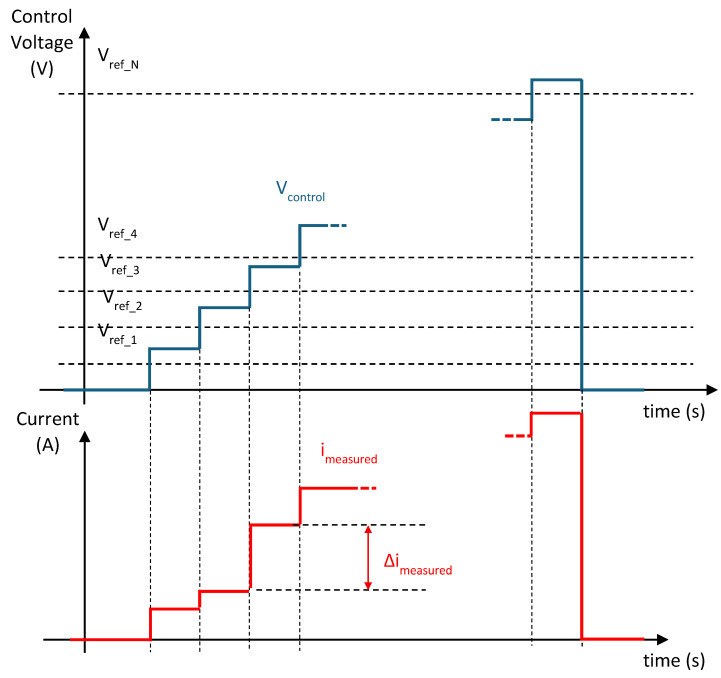
Stepwise control scheme and shape of the measured current.

The measurement principle is illustrated in Figure 8. During the acquisition cycle, the control voltage is progressively increased either as a staircase or as a continuous ramp. At any given moment, only those cells whose V_ref_i_ is lower than V_control_ will conduct. As a result, the measured voltage, V_measured_, always exhibits a stepped waveform. The height of each step depends only on the individual resistance R_i_(T) of the corresponding activated cell. Since each thermistor may be at a different temperature, the step sizes are not necessarily uniform.

To determine the individual resistance value R_i_(T) of each thermistor, a step-by-step approach can be applied. Each current step, ${∆i}_{measure\_i}$ (see Figure 8), corresponds to the current flowing through the last activated thermistor. If the steps are short enough, the self-heating of the previously activated thermistors can be neglected. Under these conditions, the resistance can be calculated as follows:

(8) $$R_{i}(T)=\frac{V_{sat}}{{∆i}_{measure\_i}}$$

The acquisition software must evaluate each current step to determine the value of each resistor. In this way, the resistance of each thermistor can be easily obtained.

It is important to note that if a shunt resistor is used directly to measure the current, instead of using the operational amplifier-based circuit with a virtual ground, the determination of each thermistor’s resistance becomes less accurate. In this case, the value of the i-th resistance depends on the previously determined resistances, as described by Equation (8), leading to a progressive accumulation of measurement error.

(9) $$R_{i}(T)=\frac{V_{sat}-V_{measure}}{i_{measure}-\frac{V_{sat}-V_{measure}}{R_{1}\left(T\right)}-\frac{V_{sat}-V_{measure}}{R_{2}\left(T\right)}-\cdots -\frac{V_{sat}-V_{measure}}{R_{i-1}(T)}}\;$$

## 3. Experimental Setup and System Calibration

### 3.1. System Implementation and Validation

**Figure 9:**
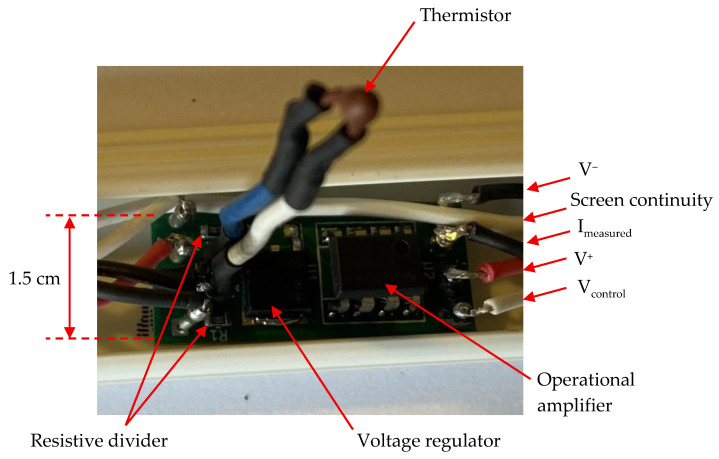
Image of the electronic circuit of each individual measurement cell.

To validate the proposed measurement method, five measurement cells were designed and assembled. Each of them is based on an NTC thermistor connected to a comparator-based switching circuit (Figure 9). The measurement cells were installed in plastic enclosures for mechanical protection.

The activation of the measurement cells is managed by applying a staircase control voltage that is progressively increased, as illustrated in Figure 8. This control voltage is generated by a National Instruments (Austin, TX, USA) USB-6008 acquisition card, which provides an analog output in the 0–5 V range. To achieve the required control levels for sequential activation, the voltage signal is amplified by a factor of 2 using a simple analog amplifier circuit housed inside a shielded box which also contains the operational amplifier used for current measurement. The USB-6008 card is also used to acquire the voltage of the shunt resistor (the output of the operational amplifier). The measurement is performed by averaging 1000 samples acquired at a rate of 10 kSamples/s. This approach yields a voltage resolution of 0.1 mV, which corresponds to a theoretical resolution of 0.02 °C in the low-temperature range (25 °C) and 0.003 °C in the high-temperature range (65 °C).

For system verification, all thermistors were individually calibrated using a Fluke 9170 temperature metrology well over the range of 15 °C to 65 °C.

The relationship between thermistor resistance and temperature can be expressed as

(10) $$R_{T}=A\cdot e^{\frac{B}{T}}\mathrm{with}\;A=R_{0}\cdot e^{\frac{-B}{T_{0}}}$$

where B is a constant of the thermistor (in Kelvin) and $R_{0}$ is the resistance at the temperature $T_{0}$ (in Kelvin), which in this case is 10 kΩ at 25 °C. This relationship can be written as

(11) $$ln\left(R_{T}\right)=ln\left(A\right)+\frac{B}{T}$$

**Figure 10:**
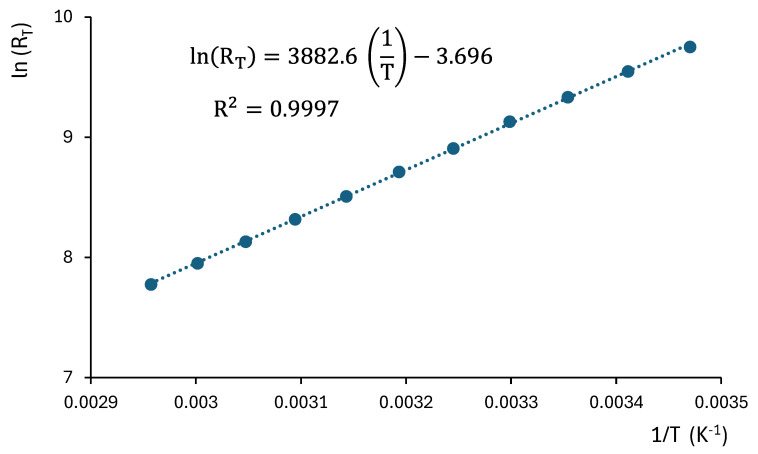
Example of calibration of measurement cell 1. The fitted constants for this cell are B = 3882.6 K and $A=e^{-3.696}=0.024822\;\Omega$.

Each measurement cell is calibrated together with the measurement circuit and the acquisition card. The coefficients of the linear Equation (11) are obtained through linear regression as illustrated in Figure 10. The parameter A is then calculated as the exponential of the constant term of the equation. The calibration results for each cell are presented in Table 1.

**Figure 11:**
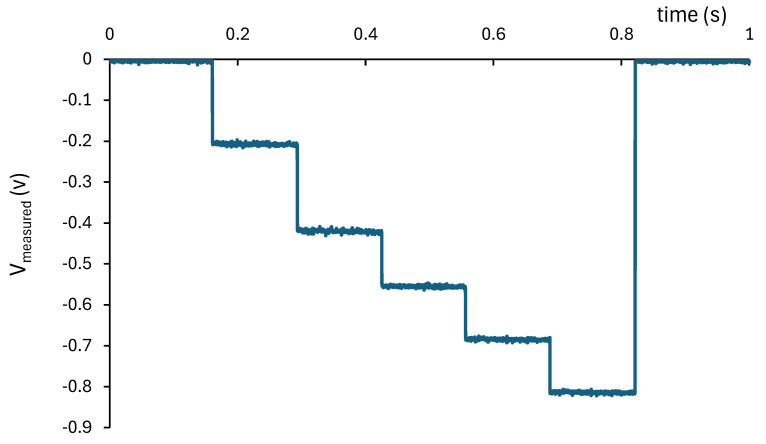
Example of measured voltage using 5 measurement cells. The first two cells were placed at 35 °C in the metrology temperature well, while the remaining three cells were at ambient temperature.

After calibration, the five measuring cells are connected in series with a spacing of 5 m between sensors and a distance of 10 m from the first sensor to the acquisition system. The absence of self-heating in the thermistors during measurement is verified by analyzing the shape and slope of each voltage step, as shown in Figure 11. Each step has a duration of 133 ms, and the maximum voltage variation per step is 3 mV. The acquisition system measures the current ($-V_{measured0}/R_{shunt}$) over a period of 100 ms at a rate of 10 kSamples/s and then calculates the average value. This acquisition time can be reduced to further to minimize self-heating of the thermistors. In any case, if the current step, ${∆i}_{measure\_i}$, is accurately determined, the influence of thermistor thermal drift on the measurement can be effectively removed.

Finally, all the sensors were placed inside an oven along with a calibrated reference thermocouple. Measurements were compared from 25 °C to 65 °C in 5 °C increments. Table 2 summarizes the results. The maximum relative error was 3.73% for cell 1 at 25 °C, while the average relative error across all measurements was 0.32%. These results indicate good agreement with the reference measurements.

### 3.2. Experimental Validation on Cables

**Figure 12:**
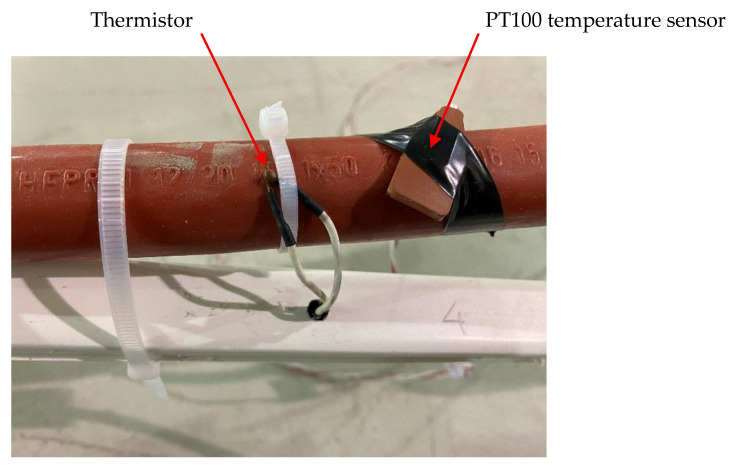
Sensor installation on the cable and reference PT-100 sensor.

**Figure 13:**
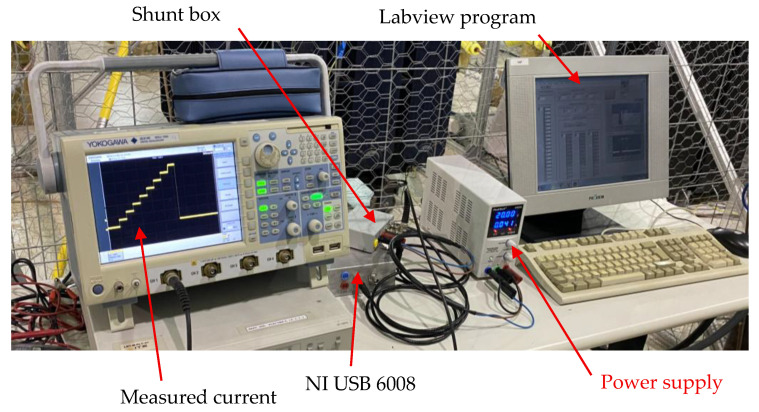
Image of the monitoring system with the computer and the NI USB 6008 card. The oscilloscope was used only for supervision of the system for a short period.

A second experimental validation was carried out on a loop where a test of thermoelectric cable ageing was conducted (Figure 12 and Figure 13). This setup was based on an earlier version of the measuring system in which a shunt resistor was used directly to measure current instead of employing the virtual ground configuration with an operational amplifier as described previously. The objective of this setup was to evaluate the system’s immunity to external magnetic fields under conditions more representative of real-world cable temperature monitoring. For this purpose, eight electronic circuits were installed on a 12/20 kV cable 50 mm^2^ aluminum conductor, mounted in open air and subjected to thermal ageing tests in four independent loops. Each loop was 20 m in length and carried a different current, all exceeding the cable’s rated current of 180 A. Consequently, the magnetic field generated was higher than in a three-phase arrangement. Nevertheless, no interference was observed in the measurements due to the 50 Hz signal.

**Figure 14:**
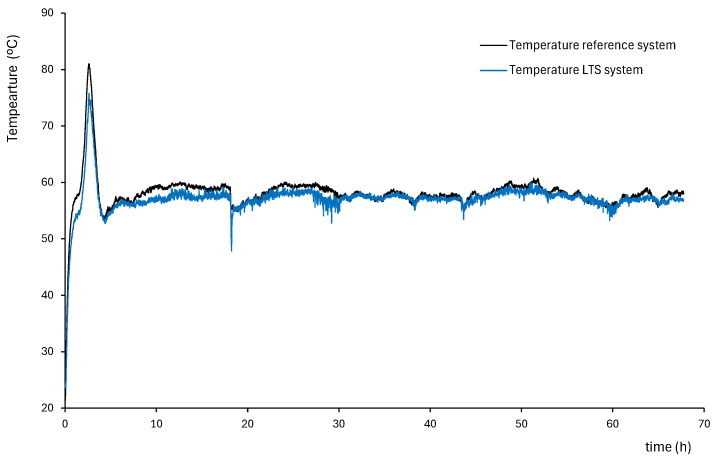
Example of temperature measurement with a commercial system and the developed LTS system at one spot.

In each loop, the measurement cell of the proposed system was installed adjacent to a commercial PT100 temperature sensor (RS Components Ltd., Corby, UK) already in use within a cable ageing setup independent of the temperature sensor development. The commercial PT100 temperature sensor served as a reference sensor. Figure 14 shows an example of a measurement point along the cable that was monitored for 70 h. The temperature peak observed in the figure corresponds to a transient current peak of 310 A, applied for 2 h as part of the cable ageing procedure. After this peak, the current was reduced and stabilized at 235 A, simulating steady-state loading conditions.

The results showed no influence from the electromagnetic field. In this preliminary version of the measurement system, an acceptable agreement between the thermistor-based measurements and those obtained using PT100 sensors was reached, with a maximum deviation of 14% observed in the thermistor readings compared to the commercial sensors. This deviation is attributed to several factors including the different positions of each sensor, their thermal contact with the sheath, the time constant of the system, and the measurement error of the LTS system due to the use of a single shunt resistor for current measurement (as described in Equation (8)). More pronounced discrepancies were observed during periods of rapid temperature variation, when the temperature distribution on the cable surface and the thermal coupling of the sensor to the cable sheath had a greater impact on measurement accuracy.

## 4. Discussion

The proposed temperature measurement system for MV underground cables offers a low-cost alternative to existing sensing technologies such as DTS systems, albeit with a reduced number of measurement points. It is also an alternative to LTS systems based on fiber optic technology and supports long-distance cable monitoring (>100 m), unlike systems based on the DS18B20 temperature sensors. The system described here uses thermistors and a sequential voltage control strategy to reduce the number of required conductors, maintaining scalability and accuracy.

Experimental results show that the system is capable of accurately determining the resistance, and thus the temperature, of each thermistor by analyzing the current steps associated with increasing V_control_. The model assumes negligible cable resistance and perfect component matching; these factors could be included in future iterations to further improve accuracy.

Only the head-end measurement system is continuously switched on. All the measurement cells are switched off during periods when temperature is not measured. There is no energy consumption by the cell or self-heating of the thermistor. For a temperature measurement cycle performed once per minute, the measurement cells may be activated for less than one second. Each current step corresponds to the contribution of the last activated thermistor. In this configuration, thermal drift due to thermistor self-heating can be removed if the current step ${∆i}_{measure\_i}$ is accurately measured.

By connecting the measurement cells in series and activating them stepwise through a common control voltage (V_control_), the system significantly minimizes the wiring complexity, using only four wires regardless of the number of measurement points. This is a critical advantage for large-scale deployment in extensive underground networks, where installation and maintenance costs are major constraints. Placement of measurement cells along a cable should be carefully decided. Representative locations of the general cable temperature should be selected for ampacity calculations and life model assessment. Additionally, critical locations including joints, terminals, and areas with ground surface changes, such as asphalt street crossings, are recommended for hotspot detection [23,24].

## 5. Conclusions

This work presents a temperature monitoring system for MV underground power cables, applicable to HVAC, HVDC, or even low-voltage power cables. The system is based on thermistors and a sequential activation strategy. The main contributions and advantages of the proposed system are as follows:

- Scalability with reduced wiring: only four wires are needed to manage an arbitrary number of temperature sensing points, enabling practical deployment in real distribution networks.
- Measurements on very long cables: these are available thanks to the use of a current loop instead of a communications protocol.
- Compact and low-power measurement cells: The use of surface-mount components allows for the integration of each cell directly on the cable with minimal size and energy consumption. The sensing cells are switched off during non-measurement periods.
- Improved accessibility: it offers an economically viable alternative to high-cost DTS systems, making continuous temperature monitoring feasible at selected locations in medium-voltage networks.

Future work will focus on experimental validation in real cable installations, improvement of the head-end measurement, mechanical reinforcement of the measurement cell, integration with existing monitoring infrastructure, and enhanced algorithms for thermal modeling and lifetime estimation based on the measured temperatures.

## Figures and Tables

**Table 1:** Constants of the calibrated measurement cells.

	B (K)	A (Ohms)
Thermistor 1	3882.574	0.024822
Thermistor 2	3927.009	0.021084
Thermistor 3	3992.663	0.016995
Thermistor 4	3947.098	0.020577
Thermistor 5	4038.138	0.014906

**Table 2:** Results of measurements in the oven.

Oven	Thermocouple	Cell 1	Cell 2	Cell 3	Cell 4	Cell 5
25 °C	26	25.03	25.82	25.39	25.57	25.39
30 °C	30.7	29.67	30.52	30.07	30.41	30.35
35 °C	35.4	34.85	35.11	35.01	35.07	35.41
40 °C	40.4	39.74	40.2	40.52	40.16	40.48
45 °C	45.5	44.94	45.18	45.66	45.06	45.67
50 °C	50.5	50.15	50.4	50.84	50.4	50.98
55 °C	55.4	55.38	55.4	55.79	56.12	56.1
60 °C	60.3	60.35	60.8	60.85	61.66	60.83
65 °C	65.3	65.72	65.73	66.35	65.85	65.74

## Data Availability

Data is available on request to the authors.

## Abbreviations

The following abbreviations are used in this manuscript:

DTS	Distributed Temperature Sensing
LTS	Localized Temperature Sensing
NTC	Negative Temperature Coefficient for Thermistors
